# Large-scale collaborative vehicle routing

**DOI:** 10.1007/s10479-021-04504-3

**Published:** 2022-04-08

**Authors:** Johan Los, Frederik Schulte, Margaretha Gansterer, Richard F. Hartl, Matthijs T. J. Spaan, Rudy R. Negenborn

**Affiliations:** 1 Department of Maritime and Transport Technology, Delft University of Technology, Mekelweg 2, 2628 CD Delft, The Netherlands; 2 Department of Operations, Energy, and Environmental Management, University of Klagenfurt, Universitätsstraße 65–67, 9020 Klagenfurt, Austria; 3 Department of Business Decisions and Analytics, University of Vienna, Oskar-Morgenstern-Platz 1, 1090 Vienna, Austria; 4 Department of Software Technology, Delft University of Technology, Van Mourik Broekmanweg 6, 2628 XE Delft, The Netherlands

**Keywords:** Collaborative vehicle routing, Combinatorial auctions, Multi-agent system, Platform-based transportation

## Abstract

Carriers can remarkably reduce transportation costs and emissions when they collaborate, for example through a platform. Such gains, however, have only been investigated for relatively small problem instances with low numbers of carriers. We develop auction-based methods for large-scale dynamic collaborative pickup and delivery problems, combining techniques of multi-agent systems and combinatorial auctions. We evaluate our approach in terms of both solution quality and possibilities of strategic behaviour using a real-world data set of over 12,000 orders. Hence, this study is (to the best of our knowledge) the first to assess the benefits of large-scale carrier cooperation and to propose an approach for it. First, we use iterative single-order auctions to investigate possible collaboration gains for increasing numbers of carriers. Our results show that travel costs can be reduced by up to 77% when 1000 carriers collaborate, largely increasing the gains that were previously observed in smaller-scale collaboration. We also ensure that individual rationality is guaranteed in each auction. Next, we compare this approach of multiple local auctions with an established central combinatorial auction mechanism and observe that the proposed approach performs better on large-scale instances. Furthermore, to improve solution quality, we integrate the two approaches by allowing small bundle auctions in the multi-agent system. We analyze the circumstances under which bundling is beneficial in a large-scale decentralized system and demonstrate that travel cost gains of up to 13% can be obtained for 1000 carriers. Finally, we investigate whether the system is vulnerable to cheating: we show that misrepresentation of true values by individual participants sometimes can benefit them at the cost of the collective. Although such strategic behaviour is not straightforward, we also discuss different means to prevent it.

## Introduction

Horizontal collaboration is an effective approach to increase transportation efficiency (Verdonck et al. 2013; Gansterer and Hartl 2018b; Pan et al. 2019) and has received increasing attention of governments, companies, and academia in the last years (Cruijssen 2020). While traditional collaborative vehicle routing focuses on exchange of orders between limited numbers of carriers, recent technological developments allow large-scale collaboration in real time. Different transportation platform companies already match orders with (partly) empty truck trips in practice, but there is a lack of academic insight into possible large-scale collaboration gains, optimization approaches, and participation incentives. This study aims to fill this gap.

Centralized collaboration approaches have been studied to assess the possible gains of collaboration (Fernández et al. 2018; Molenbruch et al. 2017; Schulte et al. 2017), but these generally make the assumption of complete control and full information availability—which generally cannot be assumed in real-world applications due to the heterogeneity of carriers and their autonomy and privacy concerns. Decentralized approaches with a central auctioneer, and combinatorial auctions in particular (Berger and Bierwirth 2010; Gansterer and Hartl 2018a), overcome these problems, but available computational studies are limited to static problems with small numbers of carriers and orders. For order allocation in larger dynamic problems, multi-agent systems (MASs) have been used, where orders are iteratively offered in auctions, and carriers place bids for them (Máhr et al. 2010; Mes et al. 2013; Los et al. 2020b). Such market-based approaches are of increasing interest: quick adjustments based on real-time data (new carriers, changing orders, schedule disturbances) are possible, without having direct control over the cooperative (but nevertheless rational) heterogeneous participants. Although there is no guarantee on optimality, its application value lies in scalability and flexibility.

In this article, we develop a MAS approach not only for allocation but also for reallocation of orders: we propose an auction-based MAS for solving large-scale dynamic collaborative pickup and delivery problems in which shippers can request transportation for their orders, and carriers can both source profitable jobs and outsource less profitable tasks. This gives us the new opportunity to examine various advantages and properties of large-scale carrier collaboration:

- First, we investigate the possible gains of cooperation among a large number of carriers. Although MASs generally have been used for allocation of orders to vehicles, they are suitable for scenarios of mere reallocation as well. Hence, we are able to examine cooperation gains on large instances with up to 1000 carriers, while such gains have only been investigated for cooperation between a few carriers so far.
- Second, we compare the performance of the MAS (consisting of multiple small iterative auctions) with the performance of (single-round, large) combinatorial auctions. Both approaches adopt limited information and decentralized control but they differ in nature. The established combinatorial auction theoretically gives the optimal solution if the auctioneer proposes all possible bundles and if all carriers give exact bids based on their individual optimal solutions. In practice, these conditions cannot be fulfilled, but good solutions can be found if the auctioneer offers a subset of well-selected bundles and the carriers use heuristics for generating bids (Gansterer and Hartl 2018a; Gansterer et al. 2020b). The MAS does not give any guarantee on optimality, but since the individual auctions are relatively cheap to perform, several subsequent reauctions might be used, which has a positive effect on solution quality (Los et al. 2020b). As an extension to our earlier paper (Los et al. 2020a), we compare both methods on instances of different size to see how they perform under different circumstances and show that the proposed MAS performs better on large-scale instances.
- Third, this paper presents a methodological contribution: to improve solution quality, we propose the integration of combinatorial aspects within the MAS. Although single-order auctions are computationally beneficial, MASs have a limited ability to deal with interaction effects of orders. Offering bundles of orders can be necessary to avoid preventable rejections, as we illustrate with the following cases. Consider two orders that are relatively close to each other, but too far from any of the available carriers to make it profitable for them to accept an individual order. If the orders are offered sequentially, none of them will be accepted. The revenue for the two orders together, however, can for some carriers be higher than the combined transportation costs, and they might gladly accept both orders when offered in a bundle. Similarly, consider two orders that have already been assigned to different carriers that each do not have capacity for combining both orders into one route, or two orders that have been assigned to the same carrier but could be served more efficiently by another carrier. In both cases, offering the orders in a bundle could cause a reallocation, while offering the individual orders in sequence might not. Hence, we expect that offering bundles within a MAS can improve results, while the extra effort for carriers to compute a bid on a bundle is limited if bundle sizes are kept very small. Thus, in this article, we extend the initial work by Los et al. (2020a) of incorporating bundles in an auction-based MAS for carrier cooperation.
- Finally, we investigate when it is beneficial for individual participants to misrepresent their true values in the developed system. In addition to Gansterer and Hartl (2018a), who show that a profitable untruthful bidding strategy is not easy to find within a combinatorial transportation auction, we theoretically show that it is not straightforward to bid strategically within the MAS. Then, we perform a computational study to show under which circumstances strategic bidding is profitable in practice in the proposed setting and discuss how we can prevent it. Thus, adding to Los et al. (2020a), we identify for the first time the possibilities of strategic behavior in local auction-based transportation collaborations.

This paper is organized as follows. In Sect. 2, we discuss the literature on collaborative approaches, and we distinguish the two trends within decentralized collaboration that we integrate within this article. Next, in Sect. 3, we describe the dynamic collaborative pickup and delivery problem that is used in our computational study. We introduce our local auction approach and propose a bundling procedure in Sect. 4. Furthermore, we recapitulate the central combinatorial auction approach that we use as a benchmark method. Section 5 presents the results from a computational study based on the data of a transportation platform company. Then, in Sect. 6, we derive the implications for companies and policy makers. Finally, Sect. 7 concludes the article and gives recommendations for future research.

## Related work

Within the field of collaborative vehicle routing, two main research areas have been distinguished: centralized collaboration and decentralized collaboration (Gansterer and Hartl 2018b, 2020).

Centralized collaboration models usually assume a set of orders for each carrier and compute what gains could theoretically be obtained if orders are exchanged. Approximation algorithms are used to compare the solution where each carrier performs only its own orders and the solution where (part of) the orders can be exchanged. It is assumed that all required information is known, which might be difficult in practice. Centralized collaboration models have been developed for different applications: Fernández et al. (2018) consider a problem where customers request service from different companies and will be attended by only a subset of these companies. Molenbruch et al. (2017) study cooperation of different dial-a-ride providers. Montoya-Torres et al. (2016) compare a non-cooperative and a cooperative scenario for a specific case of city logistics. The number of cooperating carriers in the computational studies, however, ranges from 2–4 (see Table 1). To the best of our knowledge, only Schulte et al. (2017) use larger instances of up to 50 carriers to investigate emission reductions by carrier cooperation in port-related truck operations.

A common approach for large-scale vehicle routing problems is to decompose them into smaller sub-problems that could be solved centrally. This might, however, be hard in our context, since the dynamics of newly arriving orders presumably cause a need for the subproblems’ clusters of orders to be completely revised during run time. Furthermore, decomposition approaches might be difficult when multiple carriers are involved, since not all their separate information is available. Individual carriers might, for instance, have private orders that highly influence the decomposition quality. Thus, when we want to explore large-scale collaboration in a dynamic world with hundreds or thousands of carriers that do not provide full information, we should consider decentralized collaboration approaches rather than centralized methods.

**Table 1:** Overview of collaborative transportation approaches

Cat	References	R	A	T	P	L	#Ord	#Carr	#Veh	I	B
CC	Montoya-Torres et al. (2016)	$\checkmark$					61	3	3		
	Molenbruch et al. (2017)	$\checkmark$		$\checkmark$	$\checkmark$	$\checkmark$	400	4	32		
	Schulte et al. (2017)	$\checkmark$		$\checkmark$	$\checkmark$		10–75	4–50	4–50		
	Fernández et al. (2018)	$\checkmark$				$\checkmark$	18–30	2	$\infty$		
DC	Berger and Bierwirth (2010)	$\checkmark$			$\checkmark$		$<100$	3	3		$\checkmark$
	Dai et al. (2014)	$\checkmark$		$\checkmark$	$\checkmark$	$\checkmark$	15–24	3	3–30	$\checkmark$	$\checkmark$
	Wang and Kopfer (2014)	$\checkmark$		$\checkmark$	$\checkmark$	$\checkmark$	104–266	2–5	19–61	$\checkmark$	$\checkmark$
	Li et al. (2015)	$\checkmark$			$\checkmark$	$\checkmark$	9–15	3	6	$\checkmark$	
	Wang and Kopfer (2015)	$\checkmark$		$\checkmark$	$\checkmark$		$\sim 1767$	NAv	NAv	$\checkmark$	$\checkmark$
	Lai et al. (2017)	$\checkmark$			$\checkmark$		30–245	3–24	$\infty$	$\checkmark$	
	Gansterer and Hartl (2018a)	$\checkmark$			$\checkmark$	$\checkmark$	30–210	3	3		$\checkmark$
	Lyu et al. (2019)	$\checkmark$		$\checkmark$	$\checkmark$	$\checkmark$	9–45	3	9	$\checkmark$	$\checkmark$
	Gansterer et al. (2020a)	$\checkmark$			$\checkmark$	$\checkmark$	30–90	3–6	9–18		$\checkmark$
DL	Figliozzi et al. (2004)		$\checkmark$	$\checkmark$	$\checkmark$		NAv	4	8		
	Figliozzi et al. (2005)		$\checkmark$	$\checkmark$	$\checkmark$		NAv	NAv	4		
	Máhr et al. (2010)		$\checkmark$	$\checkmark$	$\checkmark$		65	NAp	40	$\checkmark$	
	Dai and Chen (2011)	$\checkmark$		$\checkmark$	$\checkmark$	$\checkmark$	9	3	3–30	$\checkmark$	
	Mes et al. (2013)		$\checkmark$	$\checkmark$	$\checkmark$		NAv	10	10	$\checkmark$	
	Van Lon and Holvoet (2017)		$\checkmark$	$\checkmark$	$\checkmark$		120–1200	NAp	10–100	$\checkmark$	
	Los et al. (2020b)		$\checkmark$	$\checkmark$	$\checkmark$	$\checkmark$	1000	150	150	$\checkmark$	
	This article	$\checkmark$	$\checkmark$	$\checkmark$	$\checkmark$	$\checkmark$	50–2000	5–1000	5–1000	$\checkmark$	$\checkmark$

*CC* centralized collaboration, *DC* decentralized collaboration with central auctions, *DL* decentralized collaboration with local auctions, *R* reallocation of orders, *A* allocation of unassigned orders, *T* time windows, *P* pickups and deliveries, *L* less than truckload, *#Ord* number of orders, *#Carr* number of carriers, *#Veh* number of vehicles, *I* iterative auctions, *B* bundling of orders

Within the literature on decentralized collaboration, two approaches can be distinguished: decentralized collaboration with central auctions and decentralized collaboration with local auctions (see Table 1).

Decentralized collaboration with central auctions assumes that one central auctioneer interacts with all carriers but does not have complete information. An advantage is that the auctioneer can give some guarantees, e.g., it can ensure that all orders are assigned by solving the winner determination problem. The complexity of such subproblems for the coordinator, however, restricts the size of instances that can be solved. In combinatorial auctions (Berger and Bierwirth 2010; Gansterer and Hartl 2018a; Gansterer et al. 2020a), each carrier submits unprofitable orders to the auctioneer. To reduce complexity, the auctioneer proposes only a limited subset of attractive bundles of these orders, and all carriers bid on them. The auctioneer then computes the optimal assignment. Various iterative variants where bundles of orders are considered and the auctioneer finally determines a solution based on the information of different carriers have been studied by Dai et al. (2014), Lyu et al. (2019), and Wang and Kopfer (2014, 2015) (see Table 1). Other variants where bids are made only for single orders have been considered by Lai et al. (2017) and Li et al. (2015). Still, central auctions can only be applied to cooperative problem instances of limited size and are restricted to static problems. This hinders their applicability to the large-scale dynamic problem we focus on.

In decentralized collaboration with local auctions, no central auctioneer is considered. In contrast, any actor can act as auctioneer at any time by starting an auction on (part of) the order(s) that it is responsible for. Hence, local improvements can be made without guarantees on the feasibility of other orders and on global solution quality. Consequently, quick adjustments in dynamic large-scale problems are possible. Generally, this approach is used for allocation of orders to carriers (or even to separate vehicles of one carrier), but Dai and Chen (2011) apply it for reallocation as well (see Table 1). Máhr et al. (2010) and Van Lon and Holvoet (2017) consider MASs with local auctions to examine whether such a decentralized approach can outperform centralized approaches, without focusing on incentives for different carriers. Actually, they assume that all vehicles belong to a single carrier, neglecting the difficulties that arise if carriers behave competitively in their cooperations. Several carrier strategies and learning mechanisms are considered by Figliozzi et al. (2004, 2005), but they consider only full truckload problems. Mes et al. (2013) investigate the interaction of several look-ahead policies for shippers and carriers, namely delaying commitments, breaking commitments, and valuation of opportunities with respect to future orders. They, however, consider full truckload problems as well and ignore the ownership of the vehicles. Los et al. (2020b) examine the value of information sharing in a MAS, but consider allocation rather than reallocation of orders.

The present article investigates the interface between decentralized collaboration with central auctions and decentralized collaboration with local auctions: we compare both approaches and integrate them to benefit from their respective advantages when solving a complex detailed problem.

## Problem description

**Fig. 1:**
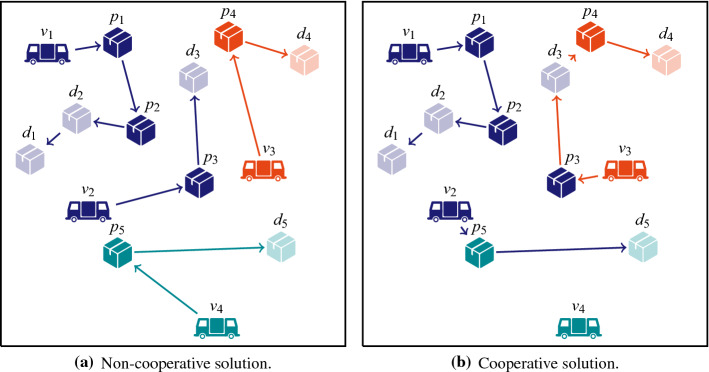
Non-cooperative and cooperative solution for an instance of a collaborative pickup and delivery problem with 3 carriers and only initially assigned orders. In the non-cooperative case, each carrier serves its own orders. In the cooperative case, travel costs can be decreased by taking over orders of other carriers

We consider a transportation platform that connects shippers and carriers, and improves routes by allowing carriers to outsource orders. We focus on a collaborative dynamic Pickup and Delivery Problem (PDP) where an order either is submitted to the platform by the shipper, or has already been assigned to a specific carrier due to a long-term contract between shipper and carrier. In the later case, the contracted carrier can be seen as the owner—the original shipper is then irrelevant. The platform organizes auctions to contract carriers for the unassigned orders. Furthermore, carriers cooperate in the sense that already contracted orders can be sold to other carriers that can deliver them cheaper (see Fig. 1).

A problem instance formally consists of a set of shippers *S*, a set of carriers *C*, a set of orders $O_s$ for each shipper $s \in S$, a set of initially assigned orders $O_c$ for each carrier $c \in C$ (with $O_S = \bigcup _{s \in S} O_s$ the total set of unassigned orders, $O_C = \bigcup _{c \in C} O_c$ the total set of assigned orders, and $O = O_S \cup O_C$ the total set of orders), and a set of capacitated vehicles $V_c$ for each carrier $c \in C$ (with $V=\bigcup _{c \in C} V_c$ the total set of vehicles).

Each order $o \in O$ represents a load of a certain quantity that must be transported from a pickup location $p_o$ to a delivery location $d_o$. The pickup or delivery, taking a certain service duration $s_i$, must start in a time window [$e_i$, $l_i$], for $i \in \{p_o, d_o\}$. The release time $r_o$ denotes when the order becomes known to the system. For $o \in O_S$, a reservation price $f_o$ is defined, i.e., a maximum value that the shipper is willing to pay for transportation.

Each vehicle $v \in V$ has an availability time window [$e_v$, $l_v$]; it becomes available at the initial location $\alpha _v$ at $e_v$ and needs to be at the end location $\omega _v$ at $l_v$. All properties of vehicles $v \in V_c$ are assumed to be known by carrier *c* at its release time $r_c$. Carriers are active from their release time until all their vehicles have become unavailable. Hence, for each time *t*, we can define the set of carriers that is known and active by $C^t = \{c \in C | r_c \le t \wedge \exists v \in V_c \ t < l_v\}$.

Travel time and travel costs from location *i* to location *j*, denoted by $t_{ij}$ and $z_{ij}$, respectively, are assumed to be identical for all vehicles throughout our computational study. In real-world cases, however, each carrier could implement its own time and cost values according to its fleet organization. The time horizon of a problem instance is denoted by $\tau$.

**Fig. 2:**
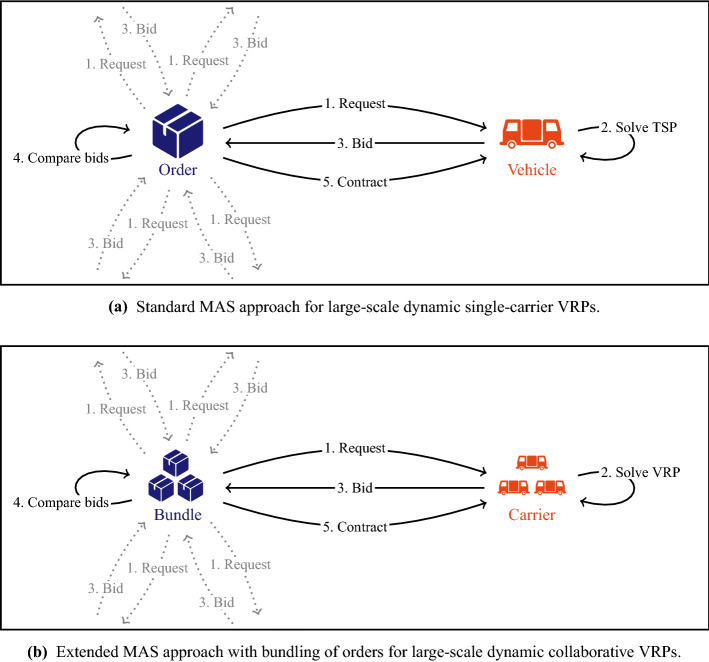
The standard MAS approach (**a**) has been extended in two ways (**b**): it is used for assignment and exchange of orders between carriers rather than for assignment to vehicles of a single carrier, and bundles are auctioned instead of single orders only

A (temporary) solution at time *t* for a problem instance is given by a set of routes $R^t = \{\langle \rho ^{1t} \rangle , \ldots , \langle \rho ^{|V|t} \rangle \}$, where each route (plan) $\langle \rho ^{vt}_i \rangle _{i=1}^{m^{vt}}$ is a sequence of $m^{vt}$ locations representing the (partially completed) path of vehicle *v* at time *t*, respecting time, capacity, and precedence constraints. A formal description of all constraints can be found in Los et al. (2020b).

Individual shippers have the goal of outsourcing their orders at a price as low as possible, but not exceeding their reservation prices. Carriers have the goal of maximizing profit, and do this by accepting and outsourcing orders such that the differences between the payment (made to them in case the order is accepted, or paid by them in case the order is outsourced) and the marginal travel costs for the orders are maximized. The individual rationality concept should be satisfied, that is, the total profits that carriers obtain through exchange of tasks should not be lower than the total profits that they obtain if they do not cooperate. Together, the goals of shippers and carriers contribute to the global goal of obtaining a final solution $R^\tau$ in which as many as possible orders are served with minimal total travel costs.

## Auction approaches

We propose a multi-agent approach where orders are iteratively offered in reverse auctions (see Fig. 2). All available carriers (acting as sellers of service) can bid for them, and the carrier with lowest bid wins the auction: it receives the price of its bid, and becomes responsible for filling the order. In contrast to previous approaches (Máhr et al. 2010; Mes et al. 2013; Los et al. 2020b), we do not restrict an auctioneer to be a shipper or carrier offering a separate order: we introduce bundle auctioneers as well, denoted by A_B_, offering a group of orders $B \subseteq O$ (see Fig. 2b). The orders within a bundle are not necessarily owned by the same shipper or carrier, since bundle auctioneers can be generated by the platform.

### Local auction procedure

When order $o \in O$ becomes available at $r_o$, auctioneer A_{o}_ (acting on behalf of shipper *s* if $o \in O_s$ or acting on behalf of carrier *c* if $o \in O_c$, but operated by the platform) is initialized and becomes active. Furthermore, the platform immediately generates, if possible, bundle auctioneers A_B_ with $o \in B$ and $|B|>1$ (based on similarity of *o* and previously released orders that are known to the platform, as will be defined in Sect. 4.2) and activates them shortly after A_{o}_ has been activated.

**Fig. 3:**
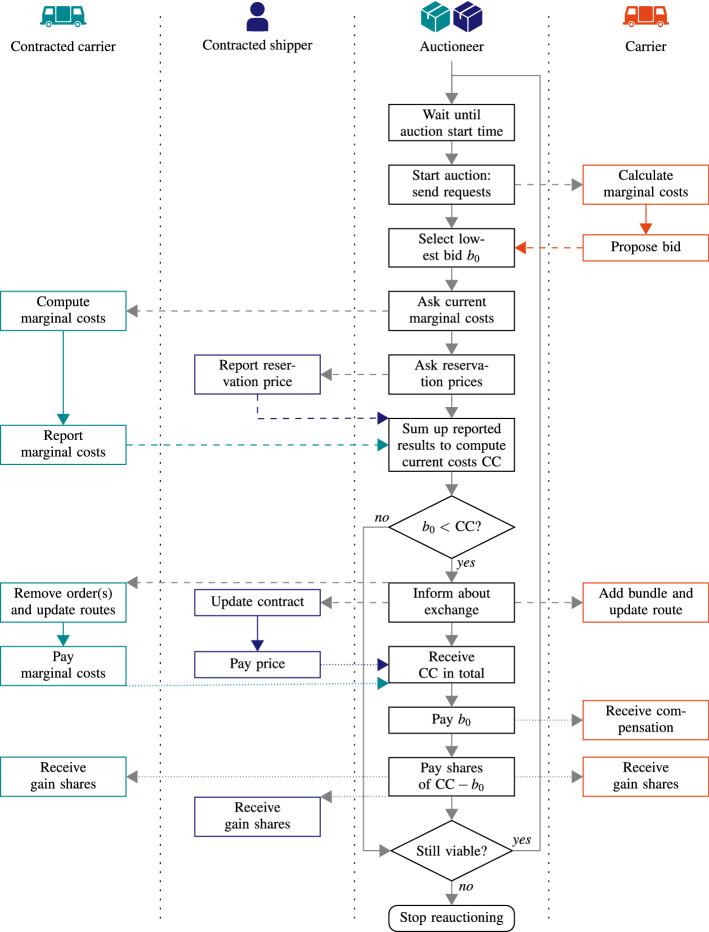
Flowchart of the iterative auction procedure within the Multi-Agent System

When active, auctioneer A_B_ repeatedly organizes auctions. Given a maximum number of auctions *a* per auctioneer and its activation time $r_{\mathrm{A}_B}$, the time between subsequent auctions is set to $(\min _{o \in B} l_{p_{o}} - r_{\mathrm{A}_B})/a$. The auction at time *t* then is as follows (see Figs. 2 and 3):

1. *Requesting transportation* A_B_ sends a request for transporting bundle *B* to all known and active carriers $c \in C^t$.
2. *Computing marginal costs* Each carrier $c \in C$ computes its individual marginal costs $\text{ MC}^t_c(B)$ for bundle *B* at time *t*, i.e., the extra travel costs for inserting all orders in *B*, according to their constraints, into its routes, given the situation at time *t*. If one or more of the orders in *B* have already been planned in the routes of the carrier, the marginal costs are computed as if these orders were not yet planned. If transporting *B* is infeasible for *c*, $\text{ MC}^t_c(B)$ is set to $\infty$.
3. *Bidding* The carriers submit a bid with value $\text{ MC}^t_c(B)$ to A_B_, i.e., they indicate that they can transport the orders if they receive at least that price.
4. *Comparing* A_B_ compares the received bids; let $b_0$ be the lowest bid provided by carrier $c_0$. Furthermore, A_B_ examines the current costs for the bundle by asking all involved carriers and shippers to report their marginal costs and reservation prices. Formally, the current costs $\text{ CC}^t(B)$ for bundle *B* at time *t* are given by the sum of the marginal costs for assigned orders and the reservation prices for unassigned orders:
  1 $$\begin{aligned} \text{ CC}^t(B) = \sum _{c \in C} \text{ MC}^t_c(B \cap O^t_c) +\sum _{o \in B \cap O^t_S} f_o, \end{aligned}$$
  where $O^t_c = \{o \in O \, | \, \exists v \in V_c \, \exists i \in \{1, \ldots , m^{vt}\} \, \rho ^{vt}_i = p_o \}$ is the total set of orders that carrier *c* has in its route plans at time *t* and $O^t_S = \{o \in O \ | \ \lnot \exists v \in V \ \exists i \in \{1, \cdots , m^{vt}\} \ \rho ^{vt}_i = p_o \}$ is the set of unassigned orders at time *t*.
5. *Updating contracts* If $b_0 < \text{ CC}^t(B)$, the bid is accepted. The platform informs all involved shippers and carriers, who update their contracts and routing plans. Furthermore, the platform receives in total $\text{ CC}^t(B)$ from the outsourcing shippers and carriers and pays $b_0$ to the winning carrier $c_0$. The gain of $\text{ CC}^t(B)-b_0$ is divided over the participants as incentive to cooperate, following some profit distribution function. Within this article, the gain is shared among the winning carrier, the (group of) currently contracted agent(s), and the platform, as defined by the following two parameters:
  *Winner Gain Share (WGS)* This parameter defines what fraction of the gain $\text{ CC}^t(B)-b_0$ is paid by the platform to the carrier winning the auction.
  *Contracted Gain Share (CGS)* This parameter defines the total fraction of the gain $\text{ CC}^t(B)-b_0$ that is paid by the platform to the currently contracted carrier(s) and/or shipper(s) for the orders within *B*. Each of them gets an equal amount.
  If WGS and CGS do not add up to 1, the remaining gains are kept by the platform. If $b_0 \ge \text{ CC}^t(B)$, no (re)allocations and no payments take place.

When transportation of one of the orders in *B* starts or the latest pickup time of one of the orders has passed without a contract for that order, A_B_ stops starting auctions and becomes inactive.

The approach guarantees that no carrier is worse off per auction, since outsourcing carriers do not pay more than their current costs for the order(s), and the winning carrier gets at least its marginal costs for the order(s). They might, however, be worse off on the long term if they get dynamically revealed yet assigned tasks that produce bad interactions with the tasks they acquired before, or that would have had good interaction effects with the tasks that they just outsourced. Nevertheless, individual rationality is guaranteed if all assigned tasks are known by the carriers beforehand, since then they can already factor in the interaction effects.

### Bundling

Selling bundles of orders within a MAS is relevant if for (some of the) individual orders, the best bid is higher than the current costs, while the best bid for the bundle is below the current costs for the bundle. This is likely to happen if orders are close to each other (both in space and time) since they might be combined within the same vehicle route with lower marginal costs.

Relatedness of orders has been defined by Ropke and Pisinger (2006) for PDPs in the context of Large Neighborhood Search (LNS). Since the goal there is to select orders from routes that can be reinserted at each other’s places, both pickup locations and delivery locations need to be similar and actual visiting times are compared. For our application, it is already sufficient if one of the locations of one order is similar to one of the locations of the other order and the time windows are not too different. Gansterer and Hartl (2018a) have investigated bundle criteria based on isolation, density and tour length. Isolation, however, is not useful in our context (since we do not require partitions of the complete set of requests) and time windows are not considered in their approach. Hence, we propose a new relatedness measure and bundling procedure that can be applied in the MAS.

We define a relatedness measure $R(o,\hat{o})$ for two orders *o* and $\hat{o}$ as follows:

2 $$\begin{aligned} R(o, \hat{o}) = \min (\text{ sim }(p_o, d_{\hat{o}}), \, \text{ sim }(d_o, p_{\hat{o}}), \, 0.5(\text{ sim } (p_o,p_{\hat{o}})+\text{ sim }(d_o,d_{\hat{o}})) ), \end{aligned}$$

where the similarity of two pickup or delivery locations *i* and *j* is defined based on both travel time and time windows:

3 $$\begin{aligned} \text{ sim }(i, j) = \gamma t_{ij} + W(i,j). \end{aligned}$$

Here, *W* represents the minimal waiting time (due to time window restrictions) at one of the locations if a vehicle serves both locations immediately after each other. Formally,

4 $$\begin{aligned} W(i,j) = \max (0, \, \min (W_D(i,j), W_D(j,i))), \end{aligned}$$

where

5 $$\begin{aligned} W_D(i,j) ={\left\{ \begin{array}{ll} \infty &{} \text{ if }\; e_i + s_i + t_{ij} > l_j;\\ \max (e_i + s_i + t_{ij}, e_j) - \min (l_i + s_i + t_{ij}, l_j) &{} \text{ otherwise }. \end{array}\right. } \end{aligned}$$

In Eq. (3), $\gamma$ is a parameter (generally $\gamma >1$) representing the cost of travel time relative to waiting time. In this article, we use $\gamma = 2$. In Eq. (2), the minimum over three terms is taken. If the pickup of one of the orders is similar to the delivery of the other order, the orders might form a good match, irrespective of the other pickup and delivery locations and times. If, however, both pickup locations are similar, it does matter whether the delivery locations are similar. If they are at opposite directions, combining the orders might appear less useful than if they are similar as well. Hence, the third term in Eq. (2) involves similarity of both pickup and delivery locations.

The platform dynamically generates bundles based on the relatedness measure *R*. Given a new order *o* at release time *t* and the pool of not yet being transported orders $O^t$, *x* bundles of size 2 and *y* bundles of size 3 are generated as follows:

- *Bundles of size 2* The platform generates bundles $\{o, {\hat{o}}\}$ for ${\hat{o}} \in O^t$ and keeps the *x* bundles with minimal $R(o,{\hat{o}})$.
- *Bundles of size 3* The platform generates bundles $\{o, \hat{o}, \check{o}\}$ for $\hat{o}, \check{o} \in O^t$ and keeps the *y* bundles for which $R(o, \hat{o}, \check{o})$ is minimal, where
  6 $$\begin{aligned} R(o, \hat{o}, \check{o}) = \min ( R(o,\hat{o})+R(\hat{o},\check{o}), \, R(o,\check{o})+R(\check{o},\hat{o}), \, R(o,\hat{o})+R(o,\check{o})). \end{aligned}$$
  Here, we have defined relatedness for three orders in such a way that not all three orders have to be highly related to each other to form an attractive bundle. Instead, each order in the bundle needs to be highly related to at least one other order in the bundle.

Throughout the experiments in this paper, we use $x = 3$ and $y=1$ to limit the computational resources needed.

### Marginal costs and route improvements

For a system dealing with dynamic reassignments, fast approximations of marginal costs are necessary. Throughout our experiments, all carriers use an elementary insertion heuristic that keeps the current sequence of orders, and inserts the new order(s) into this route at the best possible position. For bundles, the orders that can be inserted at least costs are inserted first. Hence, for a carrier $c \in C$ approximating its marginal costs for a bundle *B*, there are |*B*| main iterations in which the insertion costs for all resulting orders (at most |*B*|) at all routes ($|V_c|$ in total) are checked. Let *l* denote the current maximum vehicle route length for carrier *c*. Then insertion of both the pickup and the delivery needs to be checked for each position in the route (which can be up to $l+2|B|-2$ positions when the last order of the bundle must be inserted). Furthermore, even though we can maintain earliest and latest times along the route, a chain of time consistency updates might be necessary along the complete route in the worst case as well (Campbell and Savelsbergh 2004). Hence, the insertion heuristic has a complexity of ${\mathcal {O}}(|B|^{2} \; |V_c| \; (l+|B|)^{3})$. For single orders, this reduces to ${\mathcal {O}}(|V_c| \; l^3)$. In practice, a lot of options might be quickly pruned due to time, precedence and capacity constraints. Nevertheless, to keep computation times manageable, we limit ourselves to bundles of size 2 and 3.

To improve the quality of routes constituted by the insertion heuristic, we let carriers apply an LNS improvement phase (Pisinger and Ropke 2019) after each insertion or deletion in one of their routes. Throughout our computational study, we use the following settings. Two destroy operators, worst removal and related removal, and four repair operators, *k*-regret for $k \in \{1,2,3,4\}$, are used, as defined by Ropke and Pisinger (2006). Within each LNS iteration, a random neighbourhood size below a given maximum is selected, and a random destroy and repair operator are applied. A simple hill-climbing acceptance criterion is used, i.e., no worse solutions are accepted.

To save computation time, we do not apply the LNS improvement phase for computation of the marginal costs, but only after a bid has been accepted or an order has been outsourced. The advantage is that bids can be submitted fast. Furthermore, carriers can improve their own routes, independent of other participants, only when it is assured that a bid is accepted or an order is outsourced. Hence, they do not need to make the computational effort for each bid, with the risk of delaying the auction too much. In real-world applications, however, carriers might apply different optimization techniques, depending on the available time and resources.

### Reference approach: central combinatorial auction

To benchmark the quality of the solutions found by the MAS, we will compare it with the combinatorial auction as proposed by Gansterer et al. (2020a, 2020b). In this approach, a central auctioneer creates various sets of attractive bundles on which the carriers can bid. In contrast to the MAS, only one auction round is applied after which the auctioneer reassigns tasks to carriers. The central combinatorial auction generally consists of 5 steps (Berger and Bierwirth 2010):

1. *Request selection* Carriers can select part of their orders to submit for the auction, while they might keep other orders private.
2. *Bundling* The auctioneer creates attractive bundles of the submitted orders and opens the auction.
3. *Bidding* Carriers submit their bids, based on their marginal profits, for all bundles that they want to obtain.
4. *Winner determination* The auctioneer solves the winner determination problem, such that the total profits are maximized and each carrier obtains at most one bundle.
5. *Profit sharing* The obtained profits are shared among the participants.

In the first step, it is necessary to limit the number of submitted orders for reasons of complexity if the instance size increases. We follow the approach of Gansterer et al. (2020a), where orders that either have a low marginal profit for the carrier itself, or are expected to be attractive to other carriers are selected. To estimate potential attractiveness, all carriers provide aggregate information about the locations of their orders: a grid is superimposed upon the transport area, and each carrier provides the number of pickup and delivery locations that it has within each cell. Then, the total count by all other carriers for the two grid cells in which the pickup and delivery of an order *o* are located, indicates the attractiveness of this order to other carriers. A carrier computes for each order *o* a score, consisting of the rank of the attractiveness of *o* minus the rank of the marginal profits for *o*, and submits the orders with highest scores to the auctioneer.

Since proposing all possible bundles of submitted orders results in a too large computational load, the auctioneer applies a genetic algorithm to propose a smaller set of attractive bundles. Several partitions of the total request pool are generated. The appropriateness of a bundle is based on the distance to other bundles, the density of orders within the bundle, the minimum length of a tour visiting all the orders within the bundle, and the valuations of all carriers for the separate orders within the bundle. For details on this bundling process, we refer to Gansterer and Hartl (2018a) and Gansterer et al. (2020a).

Next, the carriers place bids consisting of their marginal costs for all offered bundles. As in Gansterer et al. (2020b), a Variable Neighborhood Search metaheuristic is applied to build the routes for the carriers.

The fourth step consists of solving the winner determination problem as described by Gansterer and Hartl (2018a). The auctioneer uses an exact approach to maximize the total profits, while each carrier is assigned at most one bundle to make sure that the solution is still feasible.

Finally, the profits of the exchange of orders can be divided over the participants in several ways (Guajardo and Rönnqvist 2016). Within the current article, however, we do not consider allocation of the profits to individuals, since we only focus on the total possible gains.

## Computational study

For our computational study, we use a real-world data set of over 12,000 orders from a Dutch transportation platform company. This company matches any submitted orders to the available load capacity of empty or partly empty trips of subscribed carriers. The data set contains locations and time windows for both pickup and delivery of each order, as well as order release times and load quantities.

To investigate possible cooperation gains (Sect. 5.1) and the impact of bundling (Sect. 5.3), we define 6 instances of 2000 orders each, and impose different assignments of orders to various numbers of carriers. To be able to compare central and local combinatorial auctions (Sect. 5.2), we define smaller instances consisting of 50–200 orders. Additionally, we use the data set provided by Gansterer and Hartl (2016) as a benchmark. In the study on strategic bidding (Sect. 5.4), we consider unassigned orders to remove any bias from unprofitable initial contracts.

### Cooperation gains

For determining possible cooperation gains in large-scale problems, we generated 6 instances with the following properties. Each instance consists of 2000 orders with pickup and delivery locations (in and close to the Netherlands) and load quantities approximately as in the original data set. Original time windows have been kept, except for shifts of whole days, such that all orders fall within a time span of 10 days. Release times have been set to the start of the time span to avoid problems with initial assignments. Per instance, 1000 identical vehicles of capacity 13.6 (loading meters) are available during the complete time span, distributed over 50 randomly chosen depots (such that each depot accommodates 20 vehicles). All vehicles are assumed to have a constant speed of 72 km/h, and Euclidean distances between all locations are used. The open problem variant is used, i.e., vehicles do not have to return to their depots after the last service. The reservation price for an order equals 1.5 times the travel costs between the pickup and delivery location.

Per instance, 5 carrier configurations (10, 50, 100, 500, or 1000 carriers) and 2 assignment configurations (close assignment or random assignment) are considered. With 10 carriers, each carrier owns 100 vehicles, i.e., precisely 5 depots. With 50 carriers, each carrier has exactly 1 depot with 20 vehicles. With 100, 500, and 1000 carriers, each carrier owns 10 vehicles, 2 vehicles, or 1 vehicle, respectively, i.e., each depot contains the vehicles of 2, 10, or 20 carriers. Each order is assigned to a depot—the depot closest to its pickup location for the close assignment configuration and a randomly selected depot for random assignment—and then randomly to a carrier having vehicles in that depot. Hence, the theoretical optimum is dependent on the carrier and assignment configurations if cooperation is not considered, but not if cooperation is considered.

**Fig. 4:**
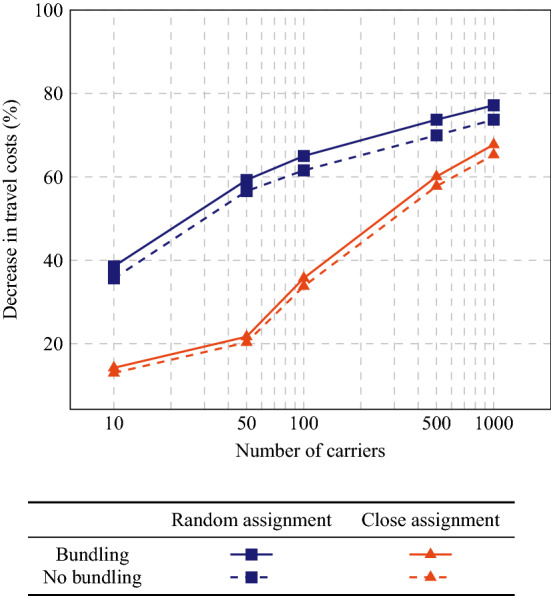
Decrease in travel costs for the cooperative scenarios (with and without bundling) compared to the non-cooperative scenario

To obtain the cooperative solutions, we apply the MAS with and without bundling three times on all instance configurations. In the runs without bundling, a maximum of 30 reauctions per order is allowed. In the runs with bundling, single orders are reauctioned a maximum of 10 times. In addition, we select the three most promising bundles of size 2 for the order and the most promising bundle of size 3 for the order (see Sect. 4.2), and auction them a maximum of 5 times each. These parameters are selected in such a way that the total number of reauctions for each order is equal with and without bundling. Note, however, that some orders might be offered more than 30 times if they appear in bundles of other orders as well. In both cases, each carrier applies a small LNS improvement phase (100 iterations, at most 5 orders per iteration) only after an auction causes an insertion or deletion in one of its routes.

To obtain the solutions of the non-cooperative scenario, we use the following procedure for each carrier. Initially, the insertion heuristic is used to include all the tasks of the carrier into the routes of its vehicles, and afterwards an LNS improvement phase of 2500 iterations with a maximum of 100 orders per iteration is applied to improve this solution. Since we have to compute this non-cooperative solution only once for each carrier, we could use a much larger LNS improvement phase than the small LNS improvement phases that are iteratively performed after each auction in the cooperative scenario.

**Fig. 5:**
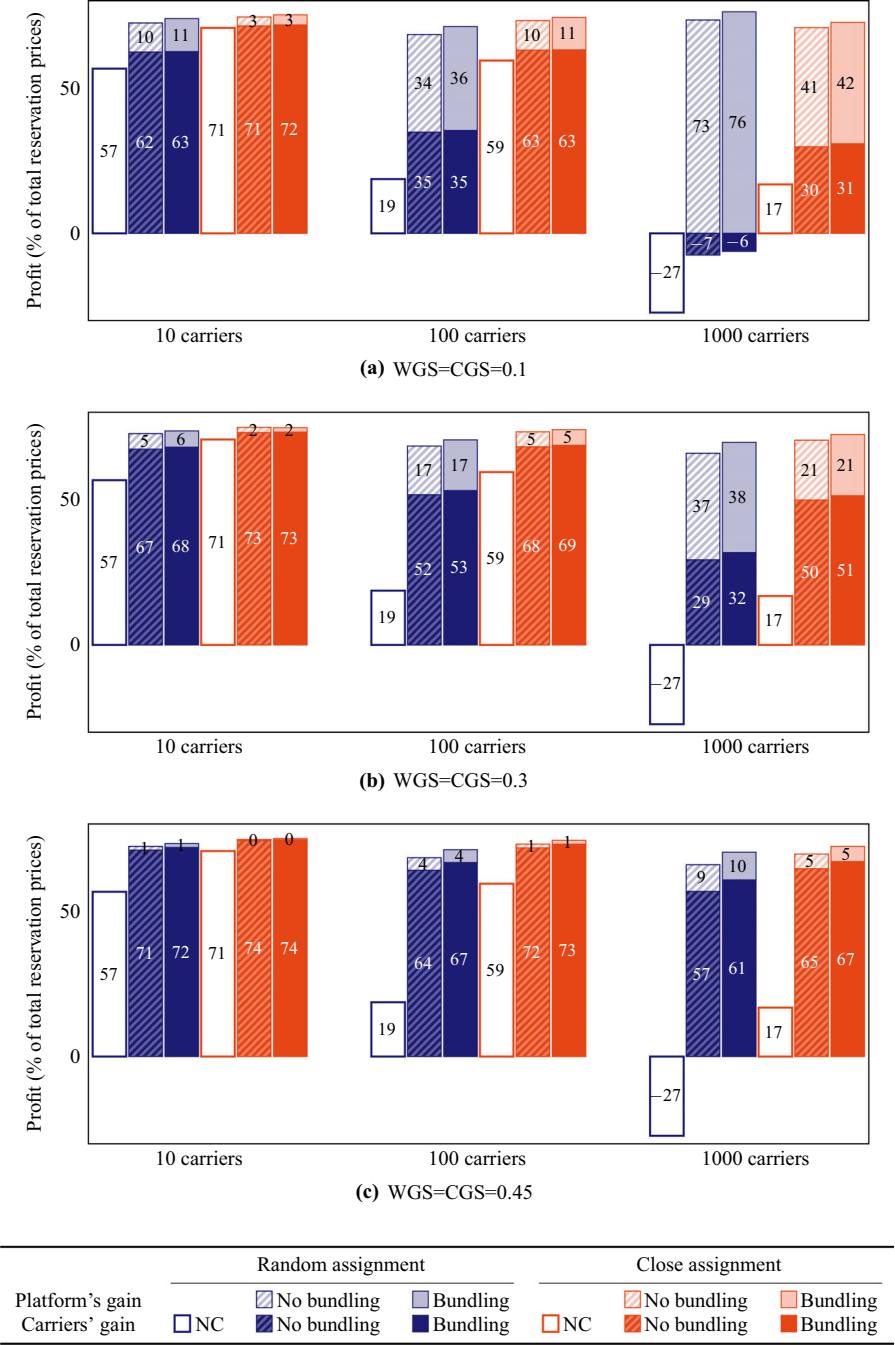
Profits for the platform and the collective of carriers on instance 1 as a percentage of the system’s revenue for different values of Winner Gain Share (WGS) and Contracted Gain Share (CGS). NC denotes the non-cooperative scenario

We show the average decrease in total travel costs for the cooperative scenarios compared to the non-cooperative scenario in Fig. 4. As expected, cooperation gains increase with the number of participating carriers, but remarkably can be as large as 77% for 1000 carriers with random assignment. Although the non-cooperative solutions with close assignment are expected to be much better than their random assignment equivalents, cooperation can also drastically reduce the travel costs for the larger instances with close assignment: we observe savings of 68% for 1000 carriers. Note that the cooperative scenarios with bundling result in higher gains than the cooperative scenarios without bundling. We will explore this in depth in Sect. 5.3. Furthermore, note that all of the 2000 orders have been accepted in all cases, except for the non-cooperative scenarios with 1000 or 500 carriers (for 1000 carriers, 2 orders on average have been rejected with random assignment and 10 orders on average with close assignment; for 500 carriers, only 2 orders on average have been rejected with close assignment). Hence, cooperation may even improve the service level.

In Fig. 5, we give an indication of profits for the platform and for the carrier collective as a fraction of the sum of all reservation prices (i.e., the total price the shippers have paid). Analogously to the gains in travel costs, the profits for both carriers and the platform increase if cooperation is applied, and slightly more with bundling than without bundling. Furthermore, the profit increases are larger when more carriers participate. Note that the exact values highly depend on the WGS and CGS parameters for larger numbers of carriers, as well as on the prices that shippers pay for transportation. Under the current settings, shippers have paid 1.5 times the travel costs from pickup to delivery locations of the orders. With random assignment among 1000 carriers, this does not compensate the high travel costs if cooperation is not allowed. With low gain shares for the carriers and shippers ($\mathrm{WGS}=\mathrm{CGS}=0.1$; Fig. 5a), carriers even make no profit after exchange of tasks (although the platform does). With higher WGS and CGS values, carriers do make profit when collaborating (Fig. 5b, c).

### Comparing central and local combinatorial auctions

Now we have seen that large cooperation gains could be obtained if we apply the MAS on large-scale instances, we naturally come to the question what the quality of the MAS itself is. Since there are no optimality guarantees, both the results for the non-cooperative and for the cooperative scenarios might differ from the optimal solutions, leaving some space for worse or even better possible cooperation gains. To get more grip on the quality of the MAS, we compare it with established methods, both on our own instances, and on a benchmark data set.

#### Company-based instances (50–200 orders)

First, we compare the MAS with the central combinatorial auction as proposed by Gansterer et al. (2020a, 2020b) (see Sect. 4.4) on instances of size varying from 50 to 200 orders. Larger problem sizes turn out to take too much time for the central combinatorial auction, unless the number of bundles would be reduced drastically. We consider 5 or 10 carriers per instance, each having their own depot. The number of vehicles equals 10% of the number of orders, and time windows are omitted, but all orders need to be done within 24 h. Other settings are equal to the settings of Sect. 5.1.

In Table 2, we show the increases in total profit by cooperation, both for the central combinatorial auction (CCA) and for the MAS with local combinatorial auctions, compared to a non-cooperative LNS solution. As expected, the CCA performs better on the smallest instances. The MAS, however, performs increasingly better when instance size increases. For the largest instances, the number of submitted orders and the total number of bundles generated wihtin the CCA needed already to be lowered to be able to solve the winner determination problem to optimality.

There is a notable difference between instances with random assignment and instances with close assignment. While the CCA finds comparable improvements for both assignments on the instances of size 100 and 200, the improvements for the MAS are much better on the instances with random assignment. An analysis of the profit values discloses that the cooperative solutions for random and close assignment instances are similar for the MAS, but different for the CCA. Hence, the CCA is much more dependent on the initial assignment than the MAS. Of course, this effect is dependent on the parameters used for the CCA, and in particular on the number of submitted orders per carrier. For the instance with 50 orders, where 5 carriers submit each at most 10 orders, the auctioneer has an almost complete view on the total set of orders, resulting in a larger improvement with random assignment.

In one case (200 orders, 5 carriers, close assignment), the MAS obtains a negative improvement. Although this appears counterintuitive, it is explainable since we did not use the non-cooperative LNS solution referred to in the table as starting point for the MAS; instead, we used the same fast LNS approximations as are used by carriers after an auction causes any change for them. These generally arrive at about 7% lower profits than the non-cooperative LNS solutions referred to in Table 2. Although the MAS compensates this in all other cases, it did not even obtain the non-cooperative solution under these specific settings. Hence, it largely depends on the parameters whether the MAS is competitive with the CCA, but in general, the MAS seems to be a reasonable alternative when the CCA suffers from scalability issues.

**Table 2:** Solution improvement due to cooperation (in terms of profit increase, relative to a non-cooperative LNS solution) for both the central combinatorial auction and the MAS with local combinatorial auctions on instances with 50–200 orders and 5–10 carriers Each row comprises the average over 10 instances

Instance properties			Central combinatorial auction			Local combinatorial auctions
Orders	Carriers	Assignment	#SO	#B	Improvement (%)	Improvement (%)
50	5	Random	10	500	**28**.**64**	22.33
50	5	Close	10	500	**12**.**62**	7.53
100	5	Random	10	500	5.19	**9**.**15**
100	5	Close	10	500	**5**.**47**	2.98
200	5	Random	10	500	1.67	**3**.**14**
200	5	Close	10	500	**1**.**76**	-1.24
200	10	Random	5	100	2.65	**12**.**80**
200	10	Close	5	100	3.52	**3**.**64**

The largest improvement in each row is given in boldface

*#SO* maximum number of submitted orders per carrier, *#B* total number of bundles generated by the auctioneer

#### Benchmark data set (30–45 orders)

Next, we apply our method on the static data set proposed by Gansterer and Hartl (2016). We benchmark against the best known solutions (BKSs) that have been found for those instances by any method, as described by Gansterer et al. (2020a, 2020b). All instances consist of 3 carriers with depots located at 200 distance units from the others. Each carrier initially has 10 (set Ox_10) or 15 (set Ox_15) orders for which the pickup and delivery locations are in a radius of 150 (set O1_xx), 200 (set O2_xx) or 300 (set O3_xx) distance units around its depot. Thus, the area of overlap is smallest for sets O1_10 and O1_15 and largest for sets O3_10 and O3_15.

**Table 3:** Average computation times for the MAS in seconds

Instance set	$\hbox {MNA}=30$		$\hbox {MNA}=300$	
	Bundling	No bundling	Bundling	No bundling
O1_10	1.20	0.51	8.26	3.89
O1_15	2.70	1.55	19.94	10.37
O2_10	1.30	0.90	8.67	4.50
O2_15	3.15	2.12	20.96	10.54
O3_10	1.76	1.34	9.40	4.73
O3_15	5.43	3.83	24.07	12.30

*MNA* maximum number of auctions per order

**Table 4:** Average maximum improvements in profit using the MAS with respect to the BKSs. The average results per instance set are calculated using the maximum profit value out of 25 runs of the MAS per instance

Instance set	$\hbox {MNA}=30$		$\hbox {MNA}=300$	
	Bundling	No bundling	Bundling	No bundling
O1_10	$-$0.31	$-$0.37	$-$0.42	$-$0.43
O1_15	$-$0.20	$-$0.30	$-$0.28	$-$0.27
O2_10	2.42	1.32	3.18	1.61
O2_15	0.63	0.41	0.75	0.44
O3_10	6.25	3.35	6.69	2.23
O3_15	2.49	1.68	2.73	1.34

*MNA* maximum number of auctions per order

**Table 5:** Average improvements in profit using the MAS with respect to the BKSs. The average results per instance set are calculated using the average profit value out of 25 runs of the MAS per instance

Instance set	$\hbox {MNA}=30$		$\hbox {MNA}=300$	
	Bundling	No bundling	Bundling	No bundling
O1_10	$-3.17$	$-3.27$	$-3.01$	$-3.12$
O1_15	$-2.51$	$-2.60$	$-2.49$	$-2.55$
O2_10	$-1.04$	$-1.92$	$-0.80$	$-1.92$
O2_15	$-2.03$	$-2.36$	$-2.05$	$-2.26$
O3_10	$-0.36$	$-1.82$	$-0.18$	$-1.82$
O3_15	$-1.21$	$-1.85$	$-1.20$	$-1.78$

*MNA* maximum number of auctions per order

We ran the MAS under standard settings (see Sect. 5.1) on those instances (except for the fact that no maximum number of orders is specified for an LNS iteration). Furthermore, we calculated the solutions where the number of allowed auctions was increased by a factor 10. For all settings, we conducted 25 runs of the algorithm. The average computation times (on an Intel Core i7-8665U CPU at 1.90 GHz; 8 cores) are given in Table 3.

For each instance, the best result out of 25 runs was used to compute the improvement *I* with respect to the BKS, given by

7 $$\begin{aligned} I = (P_{\mathrm{MAS}} - P_{\mathrm{BKS}}) / P_{\mathrm{BKS}} \times 100\%, \end{aligned}$$

where $P_{\mathrm{MAS}}$ and $P_{\mathrm{BKS}}$ denote the profit obtained by the MAS and the profit of the BKS, respectively. The average improvement per instance set is given in Table 4. For instance sets O2_10, O2_15, O3_10, and O3_15, our best solutions outperform the BKSs, with up to 6% on average for set O3_10. It should be noted, however, that the number of order exchanges might have been limited in the approaches to find the BKSs, while this was not the case with our MAS. For instance sets O1_10 and O1_15, our best solutions are slightly lower than the BKSs. We observe that allowing bundling generally results in better solutions, while allowing more auctions has a much lower impact. The detailed results provided in Tables 8, 9, and 10 in “Appendix” show that our MAS finds improvements of up to 15% on individual instances. Although we have used the best results out of 25 runs of the algorithm here, the average profits among the 25 runs are not much lower than the profits of the BKSs, as can be observed from Table 5. Thus, the MAS is competitive with the other approaches used to solve the benchmark data set, especially for the instances with large areas of customer overlap.

### Bundling benefits

We expected that applying small bundling within the MAS could improve solutions, which is further supported by the results from Fig. 4. In the following, we consider both problems in which all tasks are initially assigned, as before, and problems in which part of the orders is initially unassigned, i.e., shippers connect to the platform to find a carrier.

**Fig. 6:**
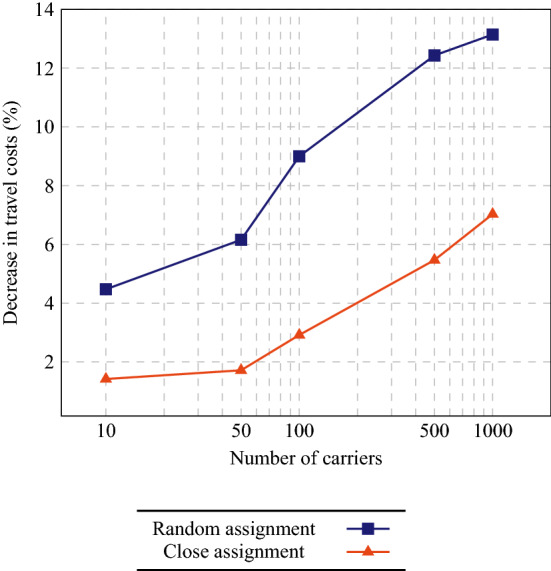
Decrease in travel costs for the bundling scenario compared to the non-bundling scenario

First, we consider the same instances as in Sect. 5.1, but now we take the scenario without bundling as base case. In Fig. 6, we show how much of the travel costs can be avoided by offering bundles. We observe that gains again increase with increasing numbers of carriers, up to 7% for 1000 carriers with close assignment and even to 13% for 1000 carriers with random assignment.

Second, we consider a more dynamic problem set in which part of the orders is not initially assigned to carriers. Again, we create 6 instances of 2000 orders each, of which only 1000 are initially assigned to carriers. We use 3 carrier configurations, namely 125, 250, or 500 carriers per instance. Each carrier has a single depot, in which it has 1–3 vehicles available. Each of the initially assigned orders is associated with a random carrier from the 10% closest carriers with respect to the pickup location. One third of the carriers have limited availability time windows, the other two third are available during the complete time span. Original order release times have been kept, except for initially assigned orders. For these, the release times equal the corresponding carrier’s release time.

We run the MAS on these instances with different numbers of carriers and various reservation price factors, both with and without bundling. The results are summarized in Table 6. The decrease in travel costs using bundling is generally between 0 and 1%, and there is a small positive influence from bundling on the service level. There is, however, no consistent pattern for increasing numbers of carriers or increasing price factors.

**Fig. 7:**
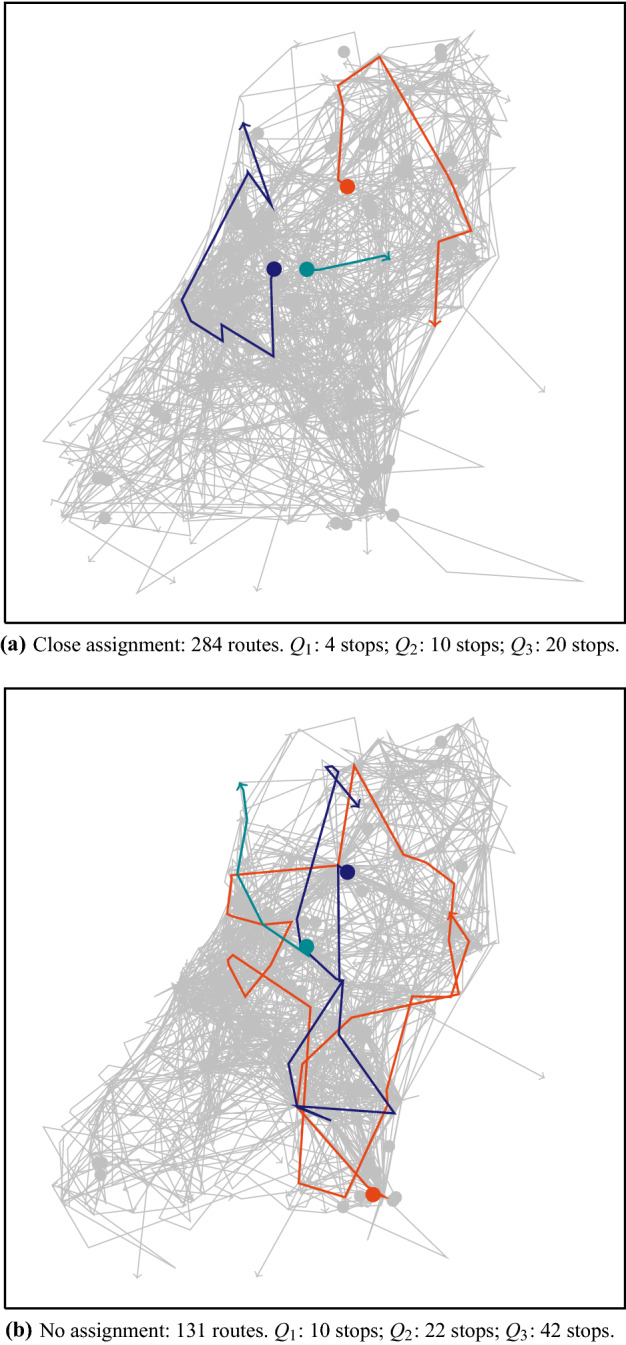
Routes for the non-cooperative scenario on instance 1 with 10 carriers, both for close assignment and no assignment. Examples of routes for the three main quartiles of length (in terms of number of stops) are highlighted in green, purple, and orange

While bundling clearly outperforms no bundling on the instances with assigned orders, it does not on the instances where part of the orders is unassigned. To explain the difference, we again consider an instance of Sect. 5.1, but remove all initial assignments. We run the MAS both with and without bundling, and define a non-cooperative scenario as well. The latter one uses in this case only 1 auction per order to get an initial assignment, followed by an LNS improvement phase by the winning carrier. In Table 7, we compare the results of these experiments to the results of the instance with initial assignment. The travel costs of the non-cooperative solution for the instance without initial assignment are generally much lower than the travel costs of the non-cooperative solution for the instances with random or close assignment. Furthermore, the number of vehicles used in the solutions for the instance without initial assignment is much lower—it is actually quite close to the final number of vehicles used in the cooperative scenarios. Hence, the average route length is larger (see Fig. 7).

This might explain the relative small difference between bundling and no bundling for the instances without initial assignment: first, the possible improvements are already lower than for instances with close or random assignment, and second, bundles of orders might be less easily accepted in longer routes, since these generally are more constrained. Note, however, that bundling still has a slight advantage on instances without initial assignment, not only in travel costs, but also in service level.

**Table 6:** Results for bundling on the partly assigned instance set where reservation prices are equal to the distance between pickup and delivery multiplied by a price factor

Carriers		Price factor		
		1.25	1.5	2
125	Average decrease in travel costs (%)	0.98	0.26	0.78
	Rejected orders with bundling (avg, [min–max])	9.22 [4–18]	3.44 [1–7]	0.56 [0–3]
	Rejected orders without bundling (avg, [min–max])	10.89 [3–17]	3.83 [1–7]	1.00 [0–3]
250	Average decrease in travel costs (%)	0.79	0.49	0.05
	Rejected orders with bundling (avg, [min–max])	6.22 [2–11]	1.50 [0–4]	0.39 [0–2]
	Rejected orders without bundling (avg, [min–max])	7.06 [3–18]	1.61 [0–4]	0.67 [0–3]
500	Average decrease in travel costs (%)	0.24	0.88	0.70
	Rejected orders with bundling (avg, [min–max])	4.67 [0–8]	1.22 [0–4]	0.61 [0–2]
	Rejected orders without bundling (avg, [min–max])	4.61 [2–7]	1.67 [0–4]	0.44 [0–2]

**Table 7:** Average results (over 3 runs) on instance 1 in terms of travel costs, service level, and used vehicles for the three scenarios. (For instances without initial assignment, the non-cooperative scenario consists of only 1 auction per order, followed by an improvement phase by the winning carrier.) *ETC (%)* extra travel costs compared to a reference LNS solution where all vehicles belong to the same carrier, *#RO* number of rejected orders (out of 2000), *#V* number of vehicles used in the solution, *NC* non-cooperative scenario, *NB* cooperative scenario without bundling, *B* cooperative scenario with bundling

Carriers	Assignment	ETC (%)			#RO			#V		
		NC	NB	B	NC	NB	B	NC	NB	B
10	Random	84.3	17.4	11.9	0.0	0.0	0.0	228	179	165
	Close	24.3	8.4	6.6	0.0	0.0	0.0	284	229	224
	No	23.0	6.2	5.8	33.0	6.7	3.3	132	125	126
100	Random	244.5	34.7	23.1	0.0	0.0	0.0	443	247	201
	Close	72.8	14.6	8.9	0.0	0.0	0.0	524	292	260
	No	26.9	6.3	6.2	27.7	5.7	4.7	138	125	125
1000	Random	438.9	44.5	26.6	5.0	0.0	0.0	862	341	246
	Close	247.8	23.1	15.2	16.3	0.0	0.0	591	235	202
	No	34.3	9.2	7.7	26.0	7.0	4.3	131	120	118

### Strategic behaviour

To get insights into the possible cooperation gains for large collaborative vehicle routing problems and the impact of bundling within a MAS, we have assumed that (estimates of the) real marginal costs are always reported. In practice, however, carriers and shippers might bid strategically to improve their individual profits. This is, however, not straightforward, as shown by Gansterer and Hartl (2018a) for central combinatorial auctions. We analyze the possible benefits of strategic behaviour within the proposed MAS, and show with a computational example that strategic bidding might be complicated.

**Fig. 8:**
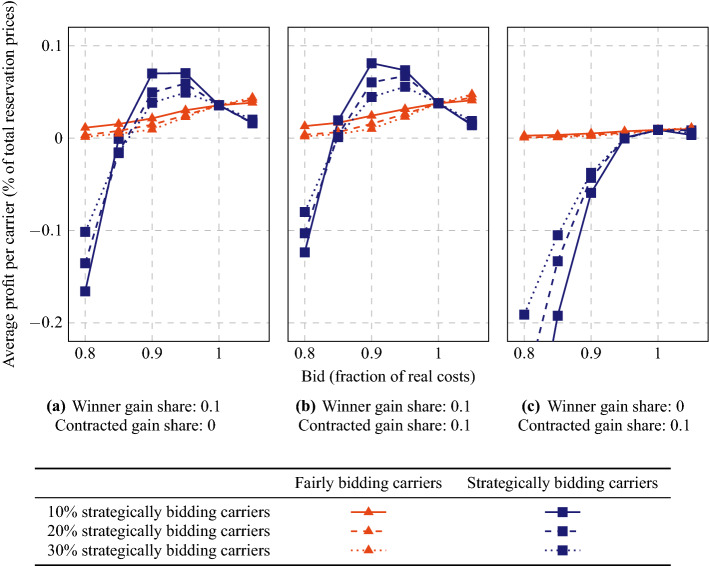
Average carrier profits if part of the carriers bid a fraction of their real (estimated) insertion costs

**Fig. 9:**
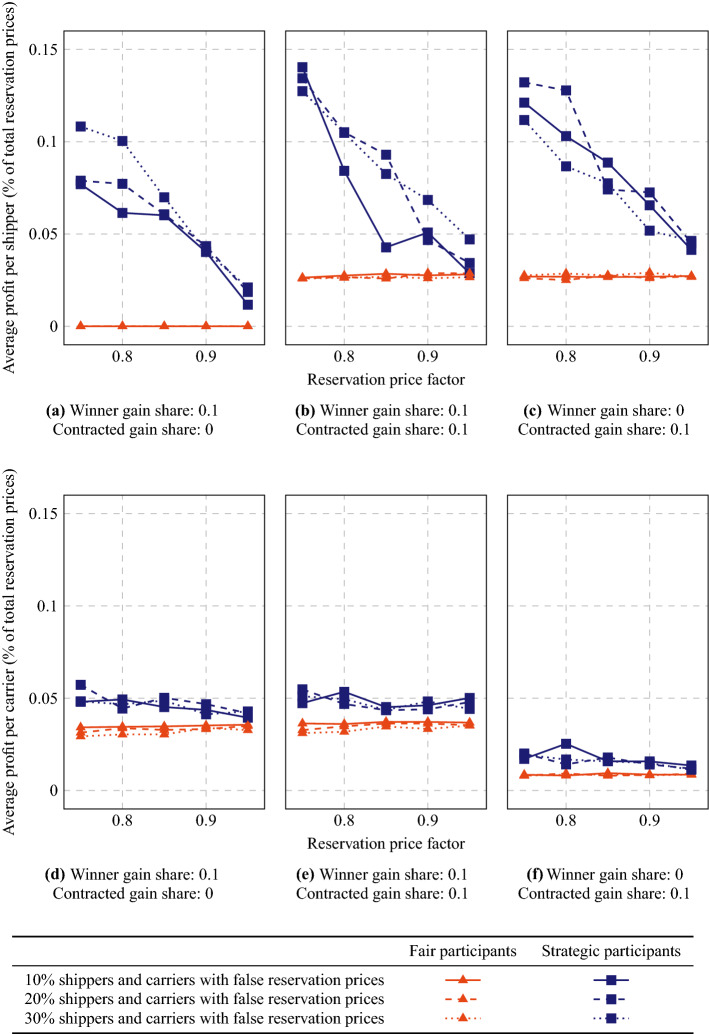
Average shipper profits (**a–c**) and carrier profits (**d–f**) if part of the shippers and carriers mention lower reservation prices than their true ones

**Fig. 10:**
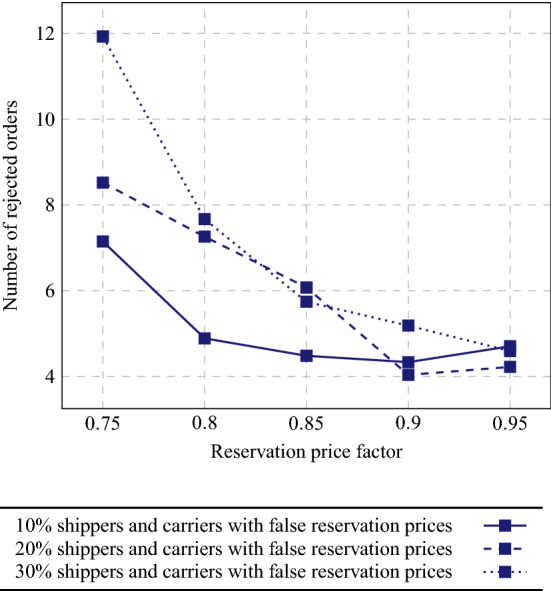
Average number of rejected orders if part of the shippers and carriers mention lower reservation prices than their true ones

For carriers placing a bid to acquire a bundle *B*, we can reason as follows, where $\text{ MC}^t_c(B)$ denotes the carrier’s marginal costs, $b_0$ denotes the carrier’s bid, and *g* denotes the profit that a winning carrier makes, i.e., *g* is a fraction of $\text{ CC}^t(B)-b_0$, dependent on the used profit distribution function.

- They will not bid a value $b_0 < \text{ MC}^t_c(B)$ if *g* is expected to be relatively small, since the compensation $b_0+g$ will not cover the extra costs $\text{ MC}^t_c(B)$.
- They might place a bid $b_0 < \text{ MC}^t_c(B)$ if *g* is expected to be relatively high. If $b_0+g > \text{ MC}^t_c(B)$, lowering the bid is a good strategy to outbid another carrier with a bid between $b_0$ and $\text{ MC}^t_c(B)$.
- They might speculate on getting a high gain from reselling the bundle later on, or foresee good interaction effects with orders that will appear later on, and hence place a bid $b_0 < \text{ MC}^t_c(B)$.
- They might bid a value $b_0 > \text{ MC}^t_c(B)$ to get a higher compensation, but this comes at the risk of not winning the auction anymore.

For carriers or shippers mentioning the marginal costs or reservation prices for outsourcing orders, we make the following observations.

- They do not report a value above their true value, since they need to pay this value.
- They might report a lower value, but this comes with the risk that the lowest bid $b_0$ is not lower than $\text{ CC}^t(B)$, hindering the trade. Indeed, they might report lower values and slightly increase them in next auction rounds, but due to the dynamic environment, there is no guarantee on success.

To prevent any bias from unprofitable initial contracts, we use problem instances without initial assignment. Again, we have 2000 orders per instance, and assume 250 carriers with 1–3 vehicles each, of which one third have restricted availability time windows. Further instance characteristics are as described before.

First, we analyze whether carriers can benefit from placing false bids. We run the MAS with different percentages of carriers (10%, 20%, or 30%) placing bids with a value of 0.8, 0.85, 0.9, 0.95, or 1.05 times their true marginal cost estimation. We test three configurations for winner gain share and contracted gain share ($\mathrm{WGS}=0.1$, $\mathrm{CGS}=0$; $\mathrm{WGS}=\mathrm{CGS}=0.1$; and $\mathrm{WGS}=0$, $\mathrm{CGS}=0.1$). In the last configuration, winning carriers do not take any of the profit generated by a succesfull auction (they even lose some profit if their bid is lower than their real costs), but they might obtain a gain if they resell the order later on.

In Fig. 8, we give the average profit per carrier, both for the fairly bidding carriers and for the strategically bidding carriers. We observe that strategic bidding pays off for a bid fraction of 0.9 or 0.95 if $\mathrm{WGS}=0.1$, but not for other bid fractions. The fairly bidding carriers are worse off if the strategic carriers bid lower than their true prices, even if the strategic carriers themselves also do not gain any extra profit. With $\mathrm{WGS}=0$ and $\mathrm{CGS}=0.1$, there is no incentive to bid another value than the true value. Note that the highest profits can be obtained if only low numbers of carriers bid strategically. Similarly, the losses that strategic carriers can obtain will be largest with low numbers of strategic carriers, i.e., if most other carriers just report their true costs. These losses can be already very large with slightly lower bid fractions. Hence, finding a beneficial bid fraction value could be a critical process. Note that with higher values for WGS, lower bid fraction values are expected to be beneficial. If the system assigns large shares of the gains to the winning carriers, cheating might appear too easy.

Next, we analyze how much shippers and carriers can benefit from communicating false (lower) reservation prices or current costs. In Fig. 9, we show average obtained profits per shipper and per carrier when 10–30% of the participants use reservation prices of 75–95% of their true values. Strategic shippers can obtain considerably high profits if they lower their communicated reservation prices. This can be explained by the large difference between reservation price and insertion costs for a carrier that already had planned a route in which the order fits quite well. The shipper then might easily outsource its order at a low price. Likewise, carriers can obtain extra profits by outsourcing orders for a lower price than their actual costs, but the differences are smaller. The drawback of using lower reservation prices, however, is that less orders will be served, as can be observed from Fig. 10.

## Policy implications

We developed a local auction system for large-scale dynamic collaborative pickup and delivery problems, and ran various experiments to investigate the advantages and disadvantages of this system. Here, we discuss the implications and limitations of the computational study.

- *Cooperation gains* Other studies generally underestimate the possible gains of cooperation due to a very small number of cooperating carriers. We found significant improvements of about 77% for 1000 collaborating carriers, but have to note that the exact savings are highly dependent on several parameters. First, the initial assignment is of importance. While the difference in improvement between the two assignments that we examined is not very large for 1000 carriers, it is more significant for lower numbers of carriers. For 50 carriers, for example, the savings with random assignment are even about three times as high as with close assignment. Second, since we compared instances with exactly the same order set, our experiments with large numbers of carriers suffer from a low number of orders per carrier. Individual routes might hence be very inefficient. Third, our approximation of the non-cooperative solutions can be too conservative. We already have seen that it performs about 7% worse than a more extensive LNS on the small instances. For larger numbers of carriers with small individual routes, however, the fast LNS approach might give a good approximation. Thus, in real-world scenarios, the benefits of cooperation can highly depend on the number of orders and on the acceptance criteria for orders that the different carriers have, as well as on their individual routing approaches. For sure, we expect a much more heterogeneous population than in our experiments.
  We showed that the profits of both the platform and the carrier collective increase with cooperation if certain percentages of the gains per transaction are given to the carriers. This may act as an incentive to participate. If the profit increases for the carriers are too low, however, they might not consider it worth the effort to cooperate. The platform is rather powerful in its decision what amount will be given to the carriers. Even if a certain share is promised, the carriers cannot verify it. Furthermore, certain individuals can significantly contribute to a better solution without receiving a significant compensation, due to the disconnected local auctions. Incentives to participate and fair profit allocations need more study, although this might be rather difficult in large-scale dynamic settings.
- *Central auctions versus local auctions* A comparison between a central combinatorial auction and an approach with local combinatorial auctions showed that the local approach is competitive with the central one for larger instance sizes. We need to emphasize, however, that both methods depend on various parameters settings. A notable finding is that the local approach is less dependent on initial assignment. What is preferable and feasible in a real-world scenario might highly depend on the specific problem properties, computational resources, and time available.
- *Bundling* Although allowing bundling within a system with unconnected local auctions can improve results by up to 13%, it is again dependent on the problem properties whether it will be useful or not. The benefits seem to be much larger when all orders have been initially assigned to carriers than in open systems where shippers still are looking for a contract. In general, however, it is advisable to use bundling, since the extra computational efforts are limited. Also, individuals may simply approximate a bid value or refuse to bid on too complex bundles if that is not feasible timewise, e.g., in a highly dynamic environment where bids need to be submitted in less than a second.
- *Strategic behaviour* We have shown that it is not straightforward to bid strategically. On the other hand, it is also not completely impossible. Carriers generally make some extra profit by lowering their bid values a little bit, but we only focused on average profits for the group of strategic carriers. Individuals might obtain losses even in this case. Furthermore, we assumed uninformed carriers, while they might make use of stochastic information about the longer term in practice.
  Moreover, we only considered a single way of distributing the gains of each auction among the participants, namely by giving them a certain share. Although more sophisticated gain sharing methods do exist (Guajardo and Rönnqvist 2016), they are intractable in large-scale settings, and are not directly applicable in decentralized approaches (Gansterer et al. 2020b). An alternative to the current approach—although it ignores the fairness property to even greater extent—could be to give a fixed financial contribution to cooperating participants, removing the direct incentives for reporting lower bids. The indirect ones might still exist, but at the same time, higher bids could be more beneficial as well.

## Conclusions and future research

Although carrier cooperation is commonly seen as a promising approach to reduce transportation costs and emissions, existing studies only show which gains can be obtained on relatively small instances. The current paper investigated the potential of large-scale transportation collaboration. Based on real-world problems, we have shown that travel cost gains of 77% can be obtained with 1000 cooperating carriers. The societal advantages in terms of emissions and traffic density are directly related. Hence, both policy makers and platform operators should provide incentives for carriers to cooperate on a larger scale.

We compared a platform-based multi-agent auction approach to a central combinatorial auction mechanism and observed that the local auction approach is competitive with the central auction if instance size increases—and even outperforms it for the largest instances. Also, the local approach is less impeded by the structure of the initial assignment. For a small-scale benchmark instance set, the local auction approach on average approximates the best known solutions, and often finds better solutions with profit improvements of up to 15%. To combine the advantages of both methods, we integrated the two approaches by allowing bundle auctions within the multi-agent system. Although the extra computational effort is limited, bundling improves the results with up to 13%.

To verify whether the proposed approach will be feasible in practice, we analyzed when strategically bidding is beneficial for individual participants. Asking lower prices than the real costs for serving an order turned out to be advantageous for carriers under certain circumstances. It is, however, highly dependent on gain shares (and hence not evident) by what amount bids can be lowered without becoming disadvantageous. On the other side, shippers or carriers outsourcing orders have an incentive to report lower reservation prices than their true ones. The drawback, however, is that a larger number of orders will not be (re)assigned. This can both hinder the improvement of the total system and harm individual shippers if their orders will not be accepted.

Future research should focus on incentive compatible mechanisms to make large-scale collaboration possible. In particular, we plan to investigate whether second-price auctions (as they are locally incentive compatible) will help in preventing strategic behaviour on the longer term, compared to the current first-price system. An interesting question in this context is whether the extra payments could be covered by bundling gains. Another relevant topic for investigation is learning from previous bids. Whereas we assumed that carriers base their bids only on their marginal costs, information of previous bids of other participants can be incorporated to improve the bidding strategy (Figliozzi et al. 2005; Mes et al. 2013; Van Heeswijk 2020). Finally, we plan to investigate a scenario with mixed levels of autonomy, where shippers and carriers either can be in charge of (re)auctioning orders themselves, or outsource this process to the platform. In this respect, the risk of being left with unassigned orders could deliberately be carried by the shipper itself, or could be transferred to the platform, that may adapt its cost structure accordingly. Trust and individual autonomy may be key conditions to fully exploit the benefits of cooperation in practice.

## Appendix: Comparison on benchmark instances

Tables 8, 9, and 10 show the results of the MAS applied to instance sets O1_10, O1_15, O2_10, O2_15, O3_10, and O3_15, and compare them with the BKSs as described by Gansterer et al. (2020a, 2020b). The profit in the MAS column is the best value among 100 runs in total (4 groups of 25 runs, with or without bundling, and with a maximum of 30 or 300 auctions per order). (Note that the results cannot be compared to the results reported by Lyu et al. (2019, Tables A14–A16), since other constraints or instance properties might have been used there. We thoroughly investigated where the differences in results might have come from, but the details of their solutions could not be found.)

**Table 8:** Best results for the MAS on benchmark set O1

Set	Instance	BKS	MAS	Improvement (%)
O1_10	run = 0 + dist = 200 + rad = 150 + n = 10	4827.75	4827.75	0.00
	run = 1 + dist = 200 + rad = 150 + n = 10	3036.25	3033.79	$-$0.08
	run = 2 + dist = 200 + rad = 150 + n = 10	3744.46	3744.46	0.00
	run = 3 + dist = 200 + rad = 150 + n = 10	3468.53	3464.23	$-$0.12
	run = 4 + dist = 200 + rad = 150 + n = 10	4286.33	4286.32	0.00
	run = 5 + dist = 200 + rad = 150 + n = 10	3686.73	3686.73	0.00
	run = 6 + dist = 200 + rad = 150 + n = 10	4599.17	4583.83	$-$0.33
	run = 7 + dist = 200 + rad = 150 + n = 10	4286.41	4286.41	0.00
	run = 8 + dist = 200 + rad = 150 + n = 10	3450.11	3489.57	1.14
	run = 9 + dist = 200 + rad = 150 + n = 10	4070.29	3969.57	$-$2.47
	run = 10 + dist = 200 + rad = 150 + n = 10	3625.60	3630.59	0.14
	run = 11 + dist = 200 + rad = 150 + n = 10	3828.01	3828.01	0.00
	run = 12 + dist = 200 + rad = 150 + n = 10	3337.18	3337.18	0.00
	run = 13 + dist = 200 + rad = 150 + n = 10	3048.79	3048.79	0.00
	run = 14 + dist = 200 + rad = 150 + n = 10	3512.80	3512.80	0.00
	run = 15 + dist = 200 + rad = 150 + n = 10	4020.28	4016.96	$-$0.08
	run = 16 + dist = 200 + rad = 150 + n = 10	3564.18	3564.18	0.00
	run = 17 + dist = 200 + rad = 150 + n = 10	4172.44	4115.91	$-$1.35
	run = 18 + dist = 200 + rad = 150 + n = 10	3945.07	4016.95	1.82
	run = 19 + dist = 200 + rad = 150 + n = 10	2885.98	2855.45	$-$1.06
	Average			$-$0.12
O1_15	run = 0 + dist = 200 + rad = 150 + n = 15	6053.44	6038.02	$-$0.25
	run = 1 + dist = 200 + rad = 150 + n = 15	6805.56	6805.56	0.00
	run = 2 + dist = 200 + rad = 150 + n = 15	6885.41	6960.50	1.09
	run = 3 + dist = 200 + rad = 150 + n = 15	7778.42	7770.54	$-$0.10
	run = 4 + dist = 200 + rad = 150 + n = 15	6079.67	6110.73	0.51
	run = 5 + dist = 200 + rad = 150 + n = 15	7594.15	7557.20	$-$0.49
	run = 6 + dist = 200 + rad = 150 + n = 15	6251.17	6323.67	1.16
	run = 7 + dist = 200 + rad = 150 + n = 15	7425.76	7408.46	$-$0.23
	run = 8 + dist = 200 + rad = 150 + n = 15	6527.29	6491.17	$-$0.55
	run = 9 + dist = 200 + rad = 150 + n = 15	6135.65	6271.66	2.22
	run = 10 + dist = 200 + rad = 150 + n = 15	6775.30	6843.83	1.01
	run = 11 + dist = 200 + rad = 150 + n = 15	7040.55	7028.88	$-$0.17
	run = 12 + dist = 200 + rad = 150 + n = 15	6389.52	6360.01	$-$0.46
	run = 13 + dist = 200 + rad = 150 + n = 15	6037.21	6021.42	$-$0.26
	run = 14 + dist = 200 + rad = 150 + n = 15	6095.78	6055.23	$-$0.67
	run = 15 + dist = 200 + rad = 150 + n = 15	6899.75	6886.70	$-$0.19
	run = 16 + dist = 200 + rad = 150 + n = 15	6525.64	6525.64	0.00
	run = 17 + dist = 200 + rad = 150 + n = 15	6217.41	6201.34	$-$0.26
	run = 18 + dist = 200 + rad = 150 + n = 15	6911.93	6911.32	$-$0.01
	run = 19 + dist = 200 + rad = 150 + n = 15	5592.14	5586.17	$-$0.11
	Average			0.11

**Table 9:** Best results for the MAS on benchmark set O2

Set	Instance	BKS	MAS	Improvement (%)
O2_10	run = 0 + dist = 200 + rad = 200 + n = 10	5681.90	6029.02	6.11
	run = 1 + dist = 200 + rad = 200 + n = 10	4105.88	4105.88	0.00
	run = 2 + dist = 200 + rad = 200 + n = 10	3323.96	3605.80	8.48
	run = 3 + dist = 200 + rad = 200 + n = 10	5840.33	5896.11	0.96
	run = 4 + dist = 200 + rad = 200 + n = 10	3923.62	4284.09	9.19
	run = 5 + dist = 200 + rad = 200 + n = 10	5172.38	5590.90	8.09
	run = 6 + dist = 200 + rad = 200 + n = 10	4306.61	4336.51	0.69
	run = 7 + dist = 200 + rad = 200 + n = 10	5113.16	5310.22	3.85
	run = 8 + dist = 200 + rad = 200 + n = 10	4415.43	4585.39	3.85
	run = 9 + dist = 200 + rad = 200 + n = 10	5474.34	5518.35	0.80
	run = 10 + dist = 200 + rad = 200 + n = 10	5314.20	5351.46	0.70
	run = 11 + dist = 200 + rad = 200 + n = 10	5351.21	5470.72	2.23
	run = 12 + dist = 200 + rad = 200 + n = 10	5778.25	5869.91	1.59
	run = 13 + dist = 200 + rad = 200 + n = 10	4885.80	5131.42	5.03
	run = 14 + dist = 200 + rad = 200 + n = 10	5217.46	5402.51	3.55
	run = 15 + dist = 200 + rad = 200 + n = 10	5518.18	5618.84	1.82
	run = 16 + dist = 200 + rad = 200 + n = 10	5431.95	5582.08	2.76
	run = 17 + dist = 200 + rad = 200 + n = 10	4566.98	4547.06	$-$0.44
	run = 18 + dist = 200 + rad = 200 + n = 10	5372.42	5624.97	4.70
	run = 19 + dist = 200 + rad = 200 + n = 10	4710.49	4881.78	3.64
	Average			3.38
O2_15	run = 0 + dist = 200 + rad = 200 + n = 15	8405.39	8459.64	0.65
	run = 1 + dist = 200 + rad = 200 + n = 15	8931.09	9090.49	1.78
	run = 2 + dist = 200 + rad = 200 + n = 15	9037.81	9054.71	0.19
	run = 3 + dist = 200 + rad = 200 + n = 15	9722.75	9994.23	2.79
	run = 4 + dist = 200 + rad = 200 + n = 15	8970.14	8948.72	$-$0.24
	run = 5 + dist = 200 + rad = 200 + n = 15	8168.25	8243.93	0.93
	run = 6 + dist = 200 + rad = 200 + n = 15	7493.37	7429.49	$-$0.85
	run = 7 + dist = 200 + rad = 200 + n = 15	7512.26	7608.29	1.28
	run = 8 + dist = 200 + rad = 200 + n = 15	6264.43	6369.51	1.68
	run = 9 + dist = 200 + rad = 200 + n = 15	7937.83	7949.73	0.15
	run = 10 + dist = 200 + rad = 200 + n = 15	7852.35	8028.10	2.24
	run = 11 + dist = 200 + rad = 200 + n = 15	8836.52	8954.58	1.34
	run = 12 + dist = 200 + rad = 200 + n = 15	9493.88	9590.01	1.01
	run = 13 + dist = 200 + rad = 200 + n = 15	8967.75	8985.83	0.20
	run = 14 + dist = 200 + rad = 200 + n = 15	7622.74	7745.15	1.61
	run = 15 + dist = 200 + rad = 200 + n = 15	9468.63	9769.13	3.17
	run = 16 + dist = 200 + rad = 200 + n = 15	8265.40	8475.67	2.54
	run = 17 + dist = 200 + rad = 200 + n = 15	8299.04	8607.71	3.72
	run = 18 + dist = 200 + rad = 200 + n = 15	9543.06	9613.84	0.74
	run = 19 + dist = 200 + rad = 200 + n = 15	8837.45	8916.38	0.89
	Average			1.29

**Table 10:** Best results for the MAS on benchmark set O3

Set	Instance	BKS	MAS	Improvement (%)
O3_10	run = 0 + dist = 200 + rad = 300 + n = 10	9032.72	10085.99	11.66
	run = 1 + dist = 200 + rad = 300 + n = 10	8336.44	8915.17	6.94
	run = 2 + dist = 200 + rad = 300 + n = 10	10150.10	10821.15	6.61
	run = 3 + dist = 200 + rad = 300 + n = 10	9075.06	9643.72	6.27
	run = 4 + dist = 200 + rad = 300 + n = 10	8650.27	9248.99	6.92
	run = 5 + dist = 200 + rad = 300 + n = 10	8359.13	8935.33	6.89
	run = 6 + dist = 200 + rad = 300 + n = 10	8555.03	9167.59	7.16
	run = 7 + dist = 200 + rad = 300 + n = 10	8580.78	9283.23	8.19
	run = 8 + dist = 200 + rad = 300 + n = 10	7960.82	8624.07	8.33
	run = 9 + dist = 200 + rad = 300 + n = 10	7328.59	7845.96	7.06
	run = 10 + dist = 200 + rad = 300 + n = 10	7491.47	8238.26	9.97
	run = 11 + dist = 200 + rad = 300 + n = 10	7242.82	7599.63	4.93
	run = 12 + dist = 200 + rad = 300 + n = 10	7223.90	8033.24	11.20
	run = 13 + dist = 200 + rad = 300 + n = 10	8095.11	9320.57	15.14
	run = 14 + dist = 200 + rad = 300 + n = 10	7750.75	8860.07	14.31
	run = 15 + dist = 200 + rad = 300 + n = 10	8304.09	8653.14	4.20
	run = 16 + dist = 200 + rad = 300 + n = 10	7780.18	8256.28	6.12
	run = 17 + dist = 200 + rad = 300 + n = 10	7022.65	7553.64	7.56
	run = 18 + dist = 200 + rad = 300 + n = 10	8462.22	8640.40	2.11
	run = 19 + dist = 200 + rad = 300 + n = 10	7733.83	7815.14	1.05
	Average			7.63
O3_15	run = 0 + dist = 200 + rad = 300 + n = 15	14156.90	14141.17	$-$0.11
	run = 1 + dist = 200 + rad = 300 + n = 15	13443.10	14067.83	4.65
	run = 2 + dist = 200 + rad = 300 + n = 15	12429.70	12839.42	3.30
	run = 3 + dist = 200 + rad = 300 + n = 15	12345.00	12755.36	3.32
	run = 4 + dist = 200 + rad = 300 + n = 15	14617.30	14635.17	0.12
	run = 5 + dist = 200 + rad = 300 + n = 15	13880.40	14520.15	4.61
	run = 6 + dist = 200 + rad = 300 + n = 15	15396.60	15709.87	2.03
	run = 7 + dist = 200 + rad = 300 + n = 15	12246.60	12813.90	4.63
	run = 8 + dist = 200 + rad = 300 + n = 15	15753.90	16234.34	3.05
	run = 9 + dist = 200 + rad = 300 + n = 15	12041.30	12411.35	3.07
	run = 10 + dist = 200 + rad = 300 + n = 15	13388.90	13960.70	4.27
	run = 11 + dist = 200 + rad = 300 + n = 15	11300.50	11507.86	1.83
	run = 12 + dist = 200 + rad = 300 + n = 15	14750.60	15332.83	3.95
	run = 13 + dist = 200 + rad = 300 + n = 15	15133.00	15822.57	4.56
	run = 14 + dist = 200 + rad = 300 + n = 15	15972.70	16632.43	4.13
	run = 15 + dist = 200 + rad = 300 + n = 15	16064.00	16617.58	3.45
	run = 16 + dist = 200 + rad = 300 + n = 15	11956.80	12309.97	2.95
	run = 17 + dist = 200 + rad = 300 + n = 15	14508.20	14923.94	2.87
	run = 18 + dist = 200 + rad = 300 + n = 15	12937.30	13505.86	4.39
	run = 19 + dist = 200 + rad = 300 + n = 15	13384.50	14179.11	5.94
	Average			3.35
